# Heterogeneous treatment effects of Cerebrolysin as an early add-on to reperfusion therapy: *post hoc* analysis of the CEREHETIS trial

**DOI:** 10.3389/fphar.2023.1288718

**Published:** 2024-01-05

**Authors:** Mikhail N. Kalinin, Dina R. Khasanova

**Affiliations:** ^1^ Department of Neurology, Kazan State Medical University, Kazan, Russia; ^2^ Department of Neurology, Interregional Clinical Diagnostic Center, Kazan, Russia

**Keywords:** Cerebrolysin, hemorrhagic transformation, functional outcome, stroke, reperfusion therapy, alteplase, heterogeneous treatment effect

## Abstract

**Background:** There has been intensive research into enhancing the effects of reperfusion therapy to mitigate hemorrhagic transformation (HT) in stroke patients. Using neuroprotective agents alongside intravenous thrombolysis (IVT) appears a promising approach. Cerebrolysin is one of the candidates since it consists of neuropeptides mimicking the action of neurotrophic factors on brain protection and repair.

**Objectives:** We looked at treatment effects of Cerebrolysin as an early add-on to IVT in stroke patients with varying HT risk.

**Methods:** It was *post hoc* analysis of the CEREHETIS trial (ISRCTN87656744). Patients with middle cerebral artery infarction (*n* = 238) were selected from the intention-to-treat population. To stratify participants according to their HT risk, the DRAGON, SEDAN and HTI scores were computed for each eligible subject using on-admission data. The study endpoints were any and symptomatic HT, and functional outcome measured with the modified Rankin Scale (mRS) on day 90. Favorable functional outcome (FFO) was defined as an mRS ≤2. The performance of each stratification tool was estimated with regression approaches. Heterogeneous treatment effect analysis was conducted using techniques of meta-analysis and the matching-smoothing method.

**Results:** The HTI score outperformed other tools in terms of HT risk stratification. Heterogeneity of Cerebrolysin treatment effects was moderate (I^2^, 35.8%–56.7%; H^2^, 1.56–2.31) and mild (I^2^, 10.9%; H^2^, 1.12) for symptomatic and any HT, respectively. A significant positive impact of Cerebrolysin on HT and functional outcome was observed in the moderate (HTI = 1) and high (HTI ≥2) HT risk patients, but it was neutral in those with the low (HTI = 0) risk. In particular, there was a steady decline in the rate of symptomatic (HTI = 0 vs. HTI = 4: by 4.3%, *p* = 0.077 vs. 21.1%, *p* < 0.001) and any HT (HTI = 0 vs. HTI = 4: by 1.2%, *p* = 0.737 vs. 32.7%, *p* < 0.001). Likewise, an mRS score reduction (HTI = 0 vs. HTI = 4: by 1.8%, *p* = 0.903 vs. 126%, *p* < 0.001) with a reciprocal increase of the fraction of FFO patients (HTI = 0 vs. HTI = 4: by 1.2% *p* = 0.757 vs. 35.5%, *p* < 0.001) was found.

**Conclusion:** Clinically meaningful heterogeneity of Cerebrolysin treatment effects on HT and functional outcome was established in stroke patients. The beneficial effects were significant in those whose estimated on-admission HT risk was either moderate or high.

## Introduction

Over the past decade, there has been intensive research into enhancing the effects of reperfusion therapy by mitigating its adverse consequences such as reperfusion injury and hemorrhagic transformation (HT), and promoting functional recovery in stroke patients (Saver, 2011). The most straightforward and promising approach appears to be the use of neuroprotective agents with multimodal pleiotropic properties along with intravenous thrombolysis (IVT) (Otsu et al., 2020). Cerebrolysin, a porcine brain derivate, is one of the candidates since it consists of low-molecular weight neuropeptides and free amino acids, which mimic the action of endogenous neurotrophic factors on protection and repair of the central nervous system (Masliah and Díez-Tejedor, 2012).

Since early 1970-s, Cerebrolysin has been widely used in treatment of acute ischemic stroke, traumatic brain injury and cognitive impairment in over 50 European, Middle East, Latin American and Asian countries but not in the United States, United Kingdom and Australia (Cui et al., 2019; Staszewski et al., 2022; Jarosz et al., 2023; Ziganshina et al., 2023). A plethora of clinical trials and meta-analyses have suggested Cerebrolysin enhances early post-stroke recovery, improves neurological deficit after stroke, and bears an excellent tolerability and safety profile (Lang et al., 2013; Guekht et al., 2017; Bornstein et al., 2018). Therefore, it has been included in the guideline on pharmacological support in motor rehabilitation after acute ischemic stroke issued by the European Academy of Neurology and the European Federation of Neurorehabilitation Societies (Beghi et al., 2021).

In our original study published in March 2023 (Khasanova and Kalinin, 2023), we looked at the effects of Cerebrolysin with IVT *versus* IVT alone in stroke patients. The rationale behind the trial came from two aspects. First, recombinant tissue plasminogen activator (rtPA) increases the HT rate by degrading the blood-brain barrier (BBB) integrity and promoting neuroinflammation and excitotoxicity (Wang et al., 2004; Kelly et al., 2006; Guan et al., 2019). On the other hand, Cerebrolysin ameliorates rtPA adverse effects and, therefore, can potentially protect from IVT-related HT (Veinbergs et al., 2000; Teng et al., 2021; Guan et al., 2019). Besides bearing neuroprotective properties directed to mitigate the reperfusion injury, Cerebrolysin also attenuates BBB permeability (Zhang et al., 2010; Teng et al., 2021), which plays an essential role in HT pathophysiology (Spronk et al., 2021).

Thus, Cerebrolysin as an early add-on to IVT turned out to be beneficial for stroke patients: the treatment was safe, and reduced the symptomatic HT rate and early neurological deficit. Furthermore, our findings were coherent with the results of a similar trial on patients with severe stroke and futile recanalization after IVT (Poljakovic et al., 2021).

However, subjects differ not only in their background characteristics, but also in how they respond to a particular treatment or intervention (Xie et al., 2012). Accordingly, it is reasonable to assume that patients’ response to the Cerebrolysin treatment would vary with respect to their HT risk stratification on admission, and hence, discovering a group of patients benefiting most by the Cerebrolysin treatment could be of paramount importance for decision making in acute stroke settings. Therefore, we conducted *post hoc* analysis of the CEREHETIS trial (Khasanova and Kalinin, 2023) to look at treatment effects of Cerebrolysin as an early add-on to the reperfusion therapy in stroke patients with varying HT risk.

## Methods

CEREHETIS was a prospective, randomized, open-label, active control, multicenter, parallel-group phase IIIb pilot study (trial registration number: ISRCTN87656744). The trial protocol, patient inclusion/exclusion criteria and original results have been published previously (Khasanova and Kalinin, 2023).

Each eligible patient was randomly assigned into either the Cerebrolysin or control group. Both arms received a standard dose of 0.9 mg/kg alteplase (Actilyse^®^, Boehringer Ingelheim GmbH, Germany), rtPA, administered intravenously within 4.5 h after symptom onset (maximal dosage 90 mg, 10% of the drug given in bolus and the rest in 60 min via intravenous infusion). Patients in the intervention group were additionally given 30 mL of Cerebrolysin^®^ (EVER Pharma GmbH, Austria) diluted in 100 mL of normal saline and infused intravenously through a separate line over 20 min. The Cerebrolysin treatment was initiated simultaneously with IVT and continued once daily for 14 consecutive days.

The study primary endpoints were the rate of any and symptomatic HT verified on a follow-up computed tomography scan from day 0 to day 14. Symptomatic HT was defined according to the ECASS III trial: any apparently extravascular blood in the brain or within the cranium that was associated with clinical deterioration, as defined by an increase of 4 points or more in the score on the National Institutes of Health Stroke Scale (NIHSS), or that led to death and that was identified as the predominant cause of the neurologic deterioration (Hacke et al., 2008).

The secondary endpoint was functional outcome measured with the modified Rankin Scale (mRS) on day 90. Favorable functional outcome (FFO) was defined as an mRS score of ≤2 on day 90.

### Study measurements

For the current study, patients with middle cerebral artery (MCA) infarction were selected from the intention-to-treat population of the CEREHETIS trial. To stratify participants according to their HT risk, several prediction tools were used: the DRAGON, SEDAN and HTI scores were computed for each eligible patient using on-admission data. By specification of each scale, a point increment corresponded to an increase in the HT probability.

Systematic reviews and meta-analyses (Whiteley et al., 2012; Sun et al., 2023) have established a number of on-admission predictors of IVT-related HT in stroke patients such as atrial fibrillation, age, serum glucose level, NIHSS, hyperdense MCA (HMCA) sign, Alberta Stroke Program Early Computed Tomography Score (ASPECTS), and onset-to-treatment time. Notwithstanding a variety of existing HT risk assessment models (Kalinin et al., 2017), our choice was restricted by several factors: an appreciation of those predictors and infarct vascular territory by each tool, effortless calculation taking into account the available dataset, external validation, and feasible use in clinical practice and trials (Fu et al., 2022).

The DRAGON score (HMCA sign/ASPECTS <10: both = 2, either = 1, none = 0; pre-stroke mRS >1: yes = 1; age: ≥80 years = 2, 65–79 years = 1, <65 years = 0; glucose: >8 mmol/L = 1; onset-to-treatment time: >90 min = 1; NIHSS: >15 = 3, 10–15 = 2, 5–9 = 1, 0–4 = 0; score range 0–10) has been designed and validated to predict functional outcome in stroke patients after IVT (Strbian et al., 2012a). However, it can also be used to assess the symptomatic HT risk since factors predicting poor functional outcome and HT are very similar (Whiteley et al., 2012).

The SEDAN score (glucose: 8.1–12.0 mmol/L = 1, >12.0 mmol/L = 2; ASPECTS <10: yes = 1; HMCA sign: yes = 1; age: >75 years = 1; NIHSS: ≥10 = 1; score range 0–6) is another well-established and externally validated tool to estimate the probability of IVT-related symptomatic HT (Strbian et al., 2012b).

The HTI score (ASPECTS: 10–7 = 0, 6–5 = 1, 4–3 = 2, 2–0 = 3; NIHSS: 0–11 = 0, 12–17 = 1, 18–23 = 2, >23 = 3; HMCA sign: yes = 1; atrial fibrillation on ECG: yes = 1; score range 0–8) has been developed and externally validated to predict any HT in MCA stroke patients regardless of IVT (Kalinin et al., 2017; Andrade et al., 2022).

### Statistical analysis

Statistical analysis was performed with Stata/MP v.14.2 and 17.0 (StataCorp LLC, United States). The descriptive statistics included median (M) with the interquartile range (IQR) for non-normally distributed continuous data and percentage for categorical data. The groups were compared with the Mann–Whitney *U* test and Pearson’s χ^2^-test for continuous and categorical variables, respectively.

Four models–Cerebrolysin treatment adjusted for the DRAGON, SEDAN, HTI score, and Cerebrolysin treatment confounded by all three scores combined–were fitted with binary logistic regression. HT (symptomatic and any) and FFO were successively defined as a dependent variable. However, only symptomatic HT was specified as a dependent variable for the assessment of postestimation statistics since its odds ratio (OR) was significantly affected by the Cerebrolysin treatment (Khasanova and Kalinin, 2023). The performance of each stratification tool was evaluated in an array of postestimation tests. Based on the results of the assessment, the best fitted model was selected for further analysis.

In order to explore a range of Cerebrolysin treatment responses in patients with varying HT risk, the chosen model was fitted with binary logistic regression followed by the assessment of conditional marginal effects. Likewise, average treatment effects (ATE), average treatment effects on the treated (ATT) and average treatment effects on the untreated (ATC) were estimated using propensity score kernel matching. In addition to the matched treatment effects, naive ATE (unconditional mean differences; without regression adjustment) (NATE) and potential outcome averages were reported (Jann, 2017).

Moreover, generalized ordered logistic regression with the proportional and partial proportional odds assumption (Williams, 2006) was applied to highlight association between the mRS score and the selected model followed by the assessment of conditional marginal effects. The parallel (proportional) odds assumption was checked with the Brant test.

Heterogeneity of Cerebrolysin treatment effects was evaluated using in-built Stata commands for meta-analysis (StataCorp, 2021). Patients were divided into several subgroups according to their HT risk score assigned by the selected model. Each subgroup was declared as a “study”. For a comparison of two-sample binary outcomes, Cerebrolysin treatment effect sizes of the “studies” measured with the risk difference were estimated in the fixed-effects Mantel–Haenszel model. Likewise, Hedges’ *g*, the standardized mean difference, was used in the fixed-effects inverse-variance model to compare two-sample continuous outcomes. The random-effects restricted maximum likelihood model was applied for both types of the outcomes with estimation of the risk difference or Hedges’ *g*, respectively. Heterogeneity statistics and a forest plot were analyzed in the output of each model. The presence of small-study effects was checked with the Egger test.

Finally, advanced heterogeneous treatment effect (HTE) analysis was performed using the matching-smoothing method (Xie et al., 2012). Cerebrolysin and the selected model were specified as a treatment and independent variable, respectively. Symptomatic and any HT, mRS, and FFO were successively defined as a dependent variable.

Following the propensity score estimation for each subject using a logit model, kernel matching was performed on the logarithm of the OR. As a result, plots of a local polynomial smooth of the treatment effect against the propensity score were generated using a local polynomial fit of degree 1 (local-linear smoothing), and the 95% confidence intervals (CIs) were estimated. The Epanechnikov kernel function was used for matching as well as for calculation of the weighted local polynomial estimate. The bandwidth was not specified, and a rule-of-thumb bandwidth estimator was calculated and used.

Furthermore, each score of the selected model was matched with the corresponding propensity score to estimate their treatment effects, and the two-tailed *p*-values were computed using the *z*-score of the 95% CIs. The HTEs included estimates from both the treated and the untreated patients.

Whenever possible, bootstrapping was performed with 1,000 samples and computing normal-based CIs to reduce sampling bias, overfitting and prediction errors. Files of Stata code (Supplementary Data Sheet S3) and raw data (Supplementary Data Sheets S1, S2) can be found in the Supplementary Material.

## Results

**FIGURE 1:**
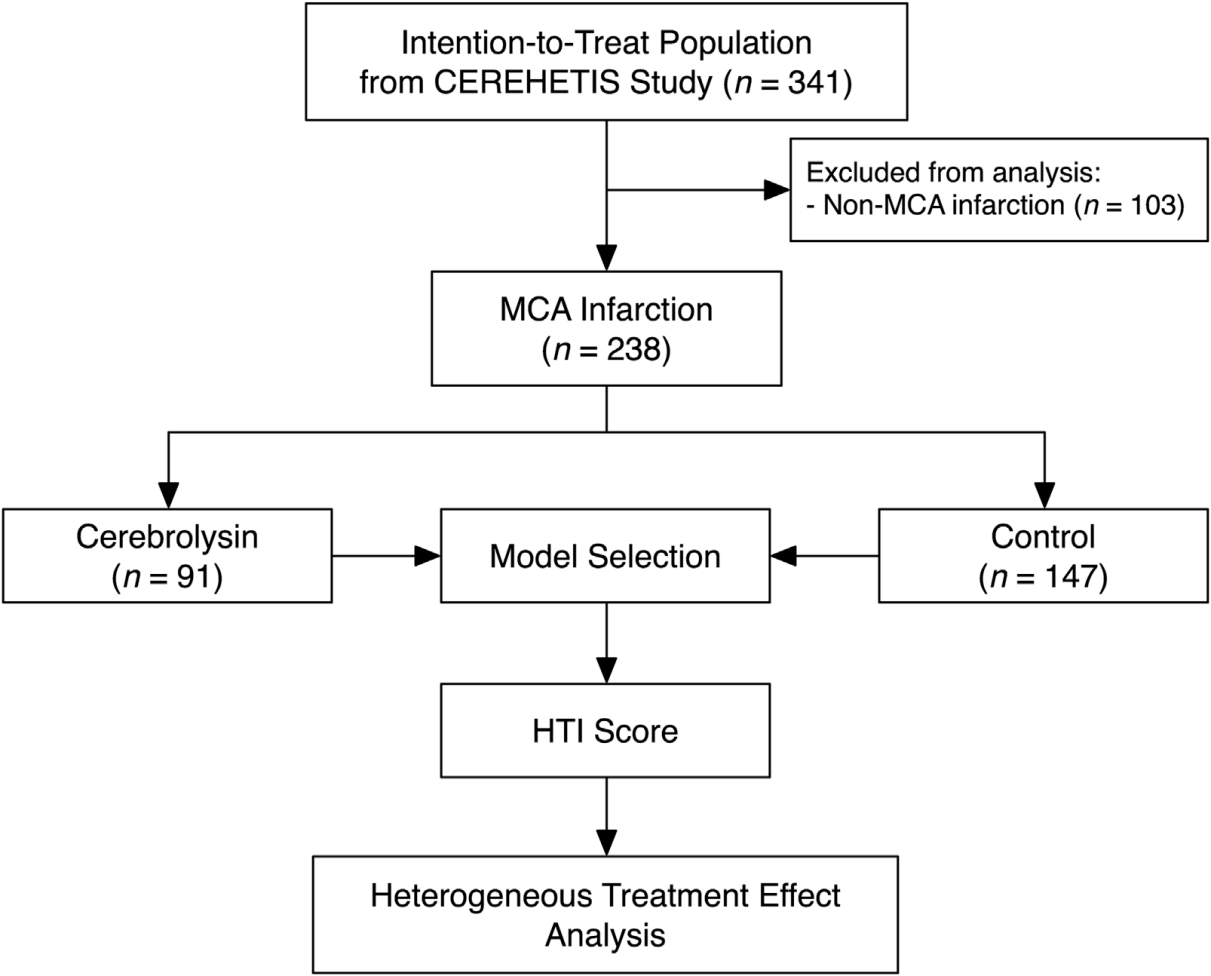
A study flowchart.

Of 341 participants comprising the intention-to-treat population of the CEREHETIS trial, 238 MCA stroke patients were selected for analysis (Figure 1).

At baseline, patients in the Cerebrolysin arm were slightly younger and, as a result, had fewer cases of previous stroke (Table 1). That was of no surprise, since the covariate disbalance had already been observed in the original cohort (Khasanova and Kalinin, 2023).

**TABLE 1:** Baseline characteristics on admission (*n* = 238).

	Cerebrolysin, *n* = 91	Control, *n* = 147	*p*-value
Age, yr (M, IQR)	64 (56–72)	69 (61–79)	0.006
Sex, male, *n* (%)	53 (58.2)	78 (53.1)	0.435
NIHSS (M, IQR)	11 (7–14)	11 (7–15)	0.687
ASPECTS (M, IQR)	10 (9–10)	10 (9–10)	0.668
HMCA sign, *n* (%)	6 (6.59)	6 (4.08)	0.389
Atrial fibrillation, history, *n* (%)	27 (29.7)	33 (22.5)	0.212
Atrial fibrillation, on ECG, *n* (%)	25 (27.5)	38 (25.9)	0.783
Diabetes mellitus, *n* (%)	17 (18.7)	22 (15.0)	0.452
Hypertension, *n* (%)	72 (79.1)	129 (87.8)	0.074
Previous stroke, *n* (%)	13 (14.3)	38 (25.9)	0.035
Previous use of antiplatelet agents, *n* (%)	24 (26.4)	39 (26.5)	0.979
Systolic blood pressure, mmHg (M, IQR)	150 (138–165)	150 (140–165)	0.618
Diastolic blood pressure, mmHg (M, IQR)	90 (80–100)	90 (80–97)	0.916
Random blood sugar, mmol/L (M, IQR)	6.5 (5.5–7.8)	6.2 (5.3–7.3)	0.254
Weight, kg (M, IQR)	80 (67–90)	74 (66–85)	0.184
Onset time, min (M, IQR)	105 (80–150)	95 (65–140)	0.295
Door-to-needle time, min (M, IQR)	40 (30–60)	40 (30–60)	0.985
Stroke subtype, *n* (%)			
Atherothrombotic	26 (28.6)	56 (38.0)	0.133
Cardioembolic	29 (31.9)	43 (29.3)	0.669
Lacunar	1 (1.1)	4 (2.7)	0.396
Other known etiology	0 (0)	1 (0.7)	0.430
Unknown etiology	35 (38.4)	43 (29.3)	0.141
Discontinue study, *n* (%)	7 (7.7)	13 (8.8)	0.756
Death	6 (6.6)	11 (7.5)	0.796
Neurosurgery	1 (1.1)	1 (0.7)	0.731
Severe medical condition	0 (0)	1 (0.7)	0.430
DRAGON score (M, IQR)	4 (3–5)	4 (3–5)	0.106
DRAGON = 0, *n* (%)	-	-	
DRAGON = 1, *n* (%)	2 (2.2)	6 (4.1)	
DRAGON = 2, *n* (%)	13 (14.3)	25 (17)	
DRAGON = 3, *n* (%)	27 (29.7)	24 (16.3)	
DRAGON = 4, *n* (%)	25 (27.5)	26 (17.7)	
DRAGON = 5, *n* (%)	13 (14.3)	38 (25.9)	
DRAGON = 6, *n* (%)	6 (6.6)	14 (9.5)	
DRAGON = 7, *n* (%)	3 (3.2)	10 (6.8)	
DRAGON = 8, *n* (%)	1 (1.1)	4 (2.7)	
DRAGON = 9, *n* (%)	1 (1.1)	-	
DRAGON = 10, *n* (%)	-	-	
SEDAN score (M, IQR)	1 (1–2)	1 (1–2)	0.879
SEDAN = 0, *n* (%)	16 (17.6)	33 (22.5)	
SEDAN = 1, *n* (%)	42 (46.2)	53 (36.1)	
SEDAN = 2, *n* (%)	20 (21.9)	35 (23.8)	
SEDAN = 3, *n* (%)	8 (8.8)	22 (14.9)	
SEDAN = 4, *n* (%)	4 (4.4)	3 (2)	
SEDAN = 5, *n* (%)	1 (1.1)	1 (0.7)	
SEDAN = 6, *n* (%)	-	-	
HTI score (M, IQR)	1 (0–2)	1 (0–2)	0.655
HTI = 0, *n* (%)	34 (37.4)	67 (45.6)	
HTI = 1, *n* (%)	34 (37.4)	37 (25.2)	
HTI = 2, *n* (%)	17 (18.7)	30 (20.4)	
HTI = 3, *n* (%)	6 (6.5)	11 (7.5)	
HTI = 4, *n* (%)	-	2 (1.3)	
HTI = 5, *n* (%)	-	-	
HTI = 6, *n* (%)	-	-	
HTI = 7, *n* (%)	-	-	
HTI = 8, *n* (%)	-	-	

ASPECTS, Alberta Stroke Program Early Computed Tomography Score; ECG, electrocardiogram; HMCA, hyperdense middle cerebral artery; IQR, interquartile range; M, median; NIHSS, National Institutes of Health Stroke Scale.

**FIGURE 2:**
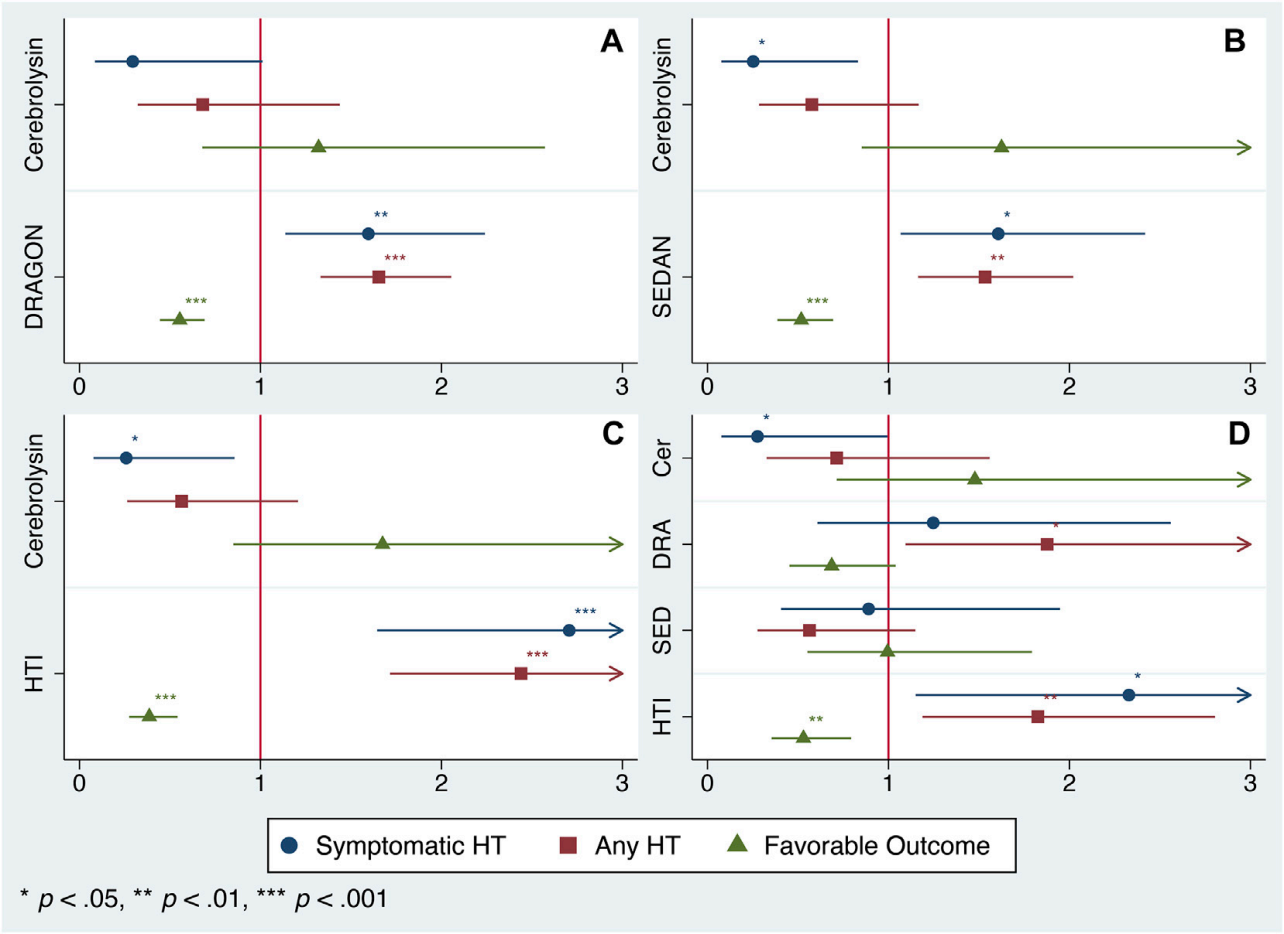
Model selection. Odds ratio (OR) of the Cerebrolysin treatment adjusted for selected scores with normal-based 95% CIs (*n* = 238). **(A)**. Model 1: OR adjusted for the DRAGON score. **(B)**. Model 2: OR adjusted for the SEDAN score. **(C)**. Model 3: OR adjusted for the HTI score. **(D)**. Model 4: OR adjusted for the DRAGON, SEDAN and HTI score combined.

Although the score distribution did not extend over the full scale for the DRAGON (0–10), SEDAN (0–6) and HTI (0–8) score (Table 1) due to the study exclusion criteria (e.g., patients with neither minor (NIHSS <4) nor severe (NIHSS >25) stroke nor high hemorrhagic risk were eligible for IVT), each tool was able to predict HT and FFO and confounded the Cerebrolysin treatment (Figures 2A–C). However, only the HTI score became significant in the combined model (Figure 2D) and its performance was superior to the competitors in the postestimation tests (Table 2), hence it was selected for further analysis.

**TABLE 2:** Postestimation statistics of the models (*n* = 238).

	Model 1	Model 2	Model 3	Model 4	Comments
Goodness of fit					
Linear predicted value squared, *p*-value	0.329	0.166	0.127	0.129	Link test for specification error, *p* > 0.05
Box-Tidwell model, *p*-value	0.705	0.426	0.519	DRAGON 0.359; SEDAN 0.937; HTI 0.412	Test for nonlinearity, *p* > 0.05
Hosmer-Lemeshow χ^2^ (df), *p*-value	4.77 (7), 0.688	5.38 (6), 0.496	4.17 (6), 0.654	7.08 (8), 0.528	Goodness of fit test, *p* > 0.05
Mean variance inflation factor	1.01	1.00	1.00	2.62	Test for collinearity, variance inflation factor <5
χ^2^					
Deviance (df)	120.96 (235)	126.36 (235)	111.78 (235)	111.09 (233)	The smaller the number the better the model fits the sample data
Likelihood ratio (df)	16.37 (2)	10.97 (2)	25.55 (2)	26.24 (4)	The bigger the number the better the model fits the sample data
*p*-value	<0.001	0.004	<0.001	<0.001	Significance of the model, *p* < 0.05
Romano-Wolf *p*-value	0.026	0.015	0.018	0.020	Significance of the model adjusted for multiple hypothesis testing, *p* < 0.05
Likelihood ratio test χ^2^ (df), *p*-value	9.87 (2), 0.007	15.27 (2), <0.001	0.69 (2), 0.708		Pairwise assumption: each model nested in Model 4; insignificance of the additional variables, *p* > 0.05
Pseudo *R* ^2^					The bigger the number the higher proportion of variance explained by the model
McFadden	0.119	0.080	0.186	0.191	
Cox-Snell	0.066	0.045	0.102	0.104	
Nagelkerke	0.152	0.103	0.232	0.238	
Information criteria					The quality estimation of each model relative to the other models; the smaller the number the better the model
AIC	126.96	132.36	117.78	121.09	
BIC (df)	137.38 (3)	142.77 (3)	128.20 (3)	138.45 (5)	
ROC-analysis					
AUC, 95% CIs	0.76 (0.67–0.84)	0.72 (0.63–0.81)	0.83 (0.75–0.90)	0.83 (0.75–0.91)	Predictive power of the model; the higher the AUC the better the model
AUC difference χ^2^ (df), *p*-value	5.10 (1), 0.024	5.75 (1), 0.017	0.13 (1), 0.719		Pairwise comparison with Model 4, *p* < 0.05

Symptomatic HT was chosen as an outcome variable. Model 1, Cerebrolysin + DRAGON score; Model 2, Cerebrolysin + SEDAN score; Model 3, Cerebrolysin + HTI score; Model 4, Cerebrolysin + DRAGON + SEDAN + HTI score; AIC, Akaike information criterion; AUC, area under ROC curve; BIC, Bayesian information criterion; CIs, confidence intervals; df, degree of freedom; ROC, receiver operating characteristic.

**FIGURE 3:**
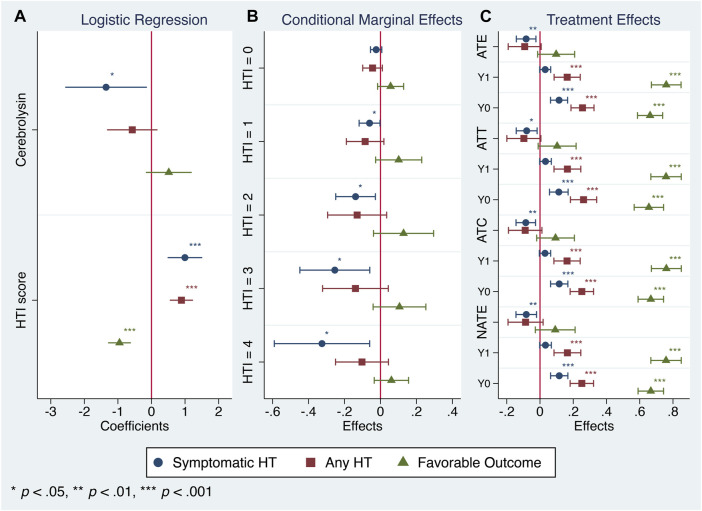
Cerebrolysin treatment responses in patients with varying HTI scores (*n* = 238). **(A)**. Logistic regression analysis. Coefficients with normal-based 95% CIs are reported. **(B)**. Conditional marginal effects of the Cerebrolysin group on probability of symptomatic HT, any HT and favorable functional outcome by the HTI score with 95% CIs. **(C)**. Treatment effects of Cerebrolysin adjusted for the HTI score with normal-based 95% CIs. Average treatment effects (ATE), average treatment effects on the treated (ATT), average treatment effects on the untreated (ATC), naive average treatment effects (NATE) and potential outcome averages (Y0–the outcome that would be obtained if a patient does not get the treatment, Y1–the outcome that would be obtained if a patient gets the treatment) are reported.

The summarized output of the employed statistical techniques–two-way contingency tables, logistic regression and propensity score matching followed by the treatment effect assessment–was consistent with our original results (Khasanova and Kalinin, 2023): the Cerebrolysin treatment significantly reduced the symptomatic HT rate and outlined a tendency to alleviate any HT and poor functional outcome (Table 3; Figures 2C, 3A, C).

**TABLE 3:** Study primary and secondary endpoints by the HTI score (*n* = 238).

	Cerebrolysin, *n* = 91	Control, *n* = 147	*p*-value
Symptomatic HT, *n* (%)	3 (3.3)	17 (11.6)	0.025
HTI = 0	0 (0)	2 (1.4)	
HTI = 1	0 (0)	4 (2.7)	
HTI = 2	1 (1.1)	7 (4.8)	
HTI = 3	2 (2.2)	3 (2)	
HTI = 4	-	1 (0.7)	
Any HT, *n* (%)	15 (16.5)	37 (25.2)	0.115
HTI = 0	2 (2.2)	5 (3.4)	
HTI = 1	6 (6.6)	11 (7.5)	
HTI = 2	4 (4.4)	13 (8.8)	
HTI = 3	3 (3.3)	7 (4.8)	
HTI = 4	-	1 (0.7)	
mRS, day 90 (M, IQR)	1 (0–2)	2 (1–3)	0.167
HTI = 0	1 (0–2)	1 (0–2)	
HTI = 1	1 (0–3)	2 (1–3)	
HTI = 2	2 (2–3)	3 (1–4)	
HTI = 3	3 (2–5)	4 (3–6)	
HTI = 4	-	4 (1–6)	
FFO, *n* (%)	69 (75.8)	98 (66.7)	0.133
HTI = 0	32 (35.2)	58 (39.5)	
HTI = 1	24 (26.4)	23 (15.6)	
HTI = 2	10 (10.9)	15 (10.2)	
HTI = 3	3 (3.3)	1 (0.7)	
HTI = 4	-	1 (0.7)	

FFO, favorable functional outcome; HT, hemorrhagic transformation; HTI, Hemorrhagic Transformation Index; IQR, interquartile range; M, median; mRS, modified Rankin Scale.

Since CEREHETIS was a prospective randomized trial with almost equal covariate distribution between the treated and the untreated (Table 1), the estimated treatment effects (ATE, ATT, ATC, NATE) and their corresponding potential outcome averages did not differ much from one another (Figure 3C), and the HTI score had no influence on the treatment assignment (propensity score matching: HTI coefficient = 0.006; 95% CI: −0.258, 0.270; *p* = 0.962).

Based on the HT predicted probability adjusted for the HTI score (Table 4), HT risk can be graded for the sake of simplicity as low (HTI = 0), moderate (HTI = 1) and high (HTI ≥2).

**TABLE 4:** Predicted probability of HT and FFO adjusted for the HTI score with 95% CIs (*n* = 248).

HTI score	Cerebrolysin, *n* = 91			Control, *n* = 147		
	Symptomatic HT	Any HT	FFO	Symptomatic HT	Any HT	FFO
0	0.009 (0.001–0.049)	0.064 (0.021–0.183)	0.901 (0.772–0.961)	0.032 (0.007–0.140)	0.108 (0.050–0.219)	0.845 (0.714–0.923)
1	0.023 (0.005–0.099)	0.144 (0.060–0.304)	0.779 (0.610–0.888)	0.084 (0.032–0.206)	0.229 (0.141–0.349)	0.678 (0.547–0.786)
2	0.060 (0.013–0.245)	0.290 (0.136–0.516)	0.576 (0.358–0.769)	0.199 (0.093–0.375)	0.420 (0.264–0.594)	0.448 (0.289–0.619)
3	0.148 (0.023–0.562)	0.499 (0.234–0.766)	0.344 (0.138–0.632)	0.402 (0.161–0.702)	0.639 (0.380–0.836)	0.239 (0.098–0.474)
4	0.320 (0.036–0.856)	0.709 (0.342–0.919)	0.168 (0.040–0.495)	0.645 (0.222–0.921)	0.812 (0.492–0.951)	0.108 (0.026–0.350)

CIs, confidence intervals; FFO, favorable functional outcome; HT, hemorrhagic transformation. Binary logistic regression followed by marginal effects estimation was used. The confidence intervals were corrected for multiple comparisons with the Šidák method.

In that respect, the assessment of conditional marginal effects of the Cerebrolysin treatment on probability of symptomatic HT by the HTI score revealed a clear pattern–the impact was neutral in the low HT risk patients but became prominent in those with the moderate and high risk. Accordingly, the Cerebrolysin treatment decreased the symptomatic HT rate by 2.4% (*p* > 0.05) in patients with HTI = 0, which further dropped step-wise to 32.5% (*p* < 0.05) at HTI = 4 (Figure 3B).

**FIGURE 4:**
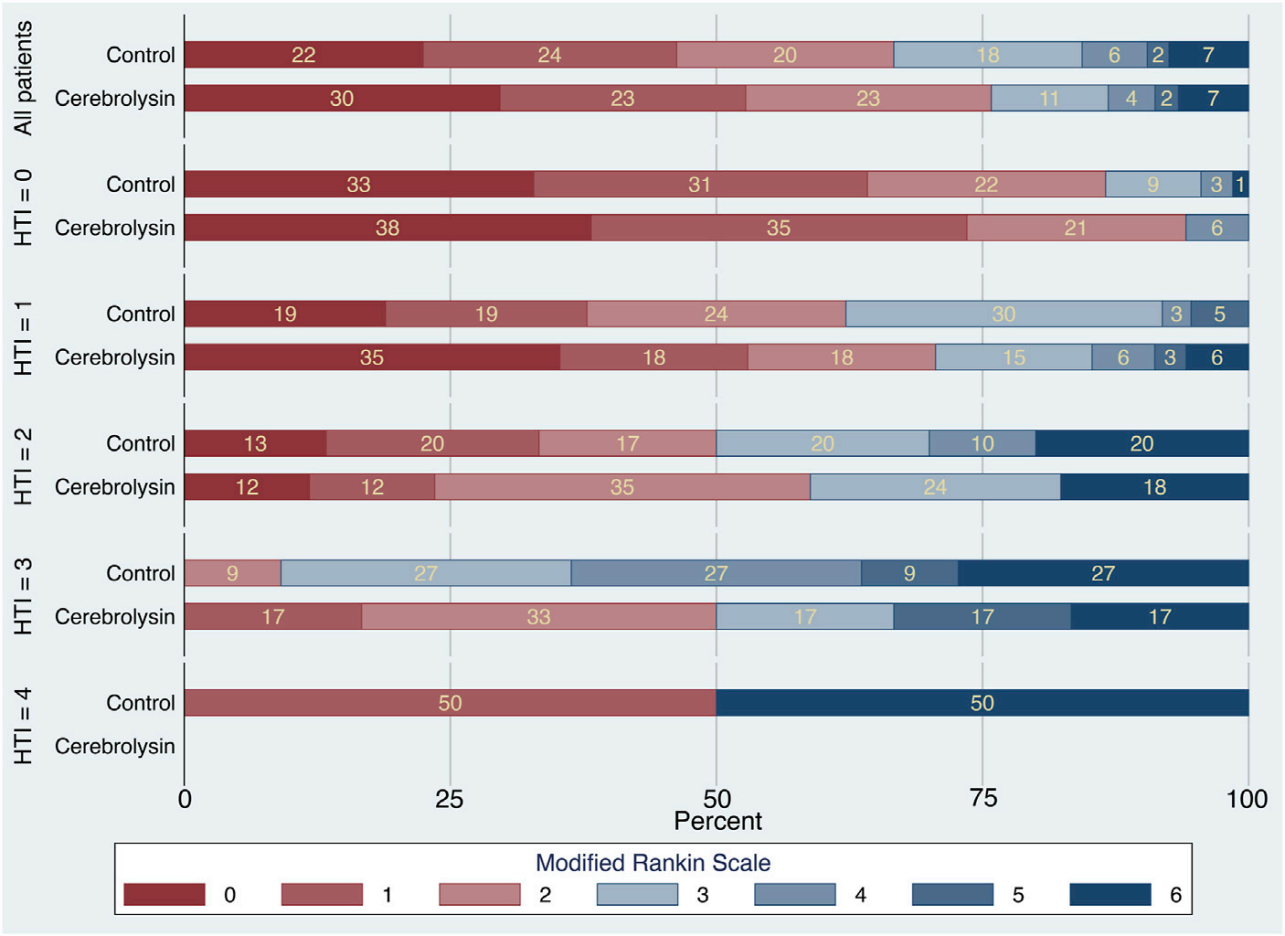
Modified Rankin Scale by the HTI score (*n* = 238).

The mRS stacked bar plot arranged by the HTI score demonstrated two positive trends in functional outcome of the Cerebrolysin group. First, at HTI = 0 and 1, there was a decrease in the percentage of patients with mRS = 3 on day 90 (HTI = 0: 0% in the Cerebrolysin arm vs. 9% in the control; HTI = 1: 15% vs. 30%, respectively). Second, the overall percentage of patients with FFO was higher in the Cerebrolysin group. Interestingly, it was more prominent at HTI = 3 (50% vs. 9%, respectively) (Figure 4).

**FIGURE 5:**
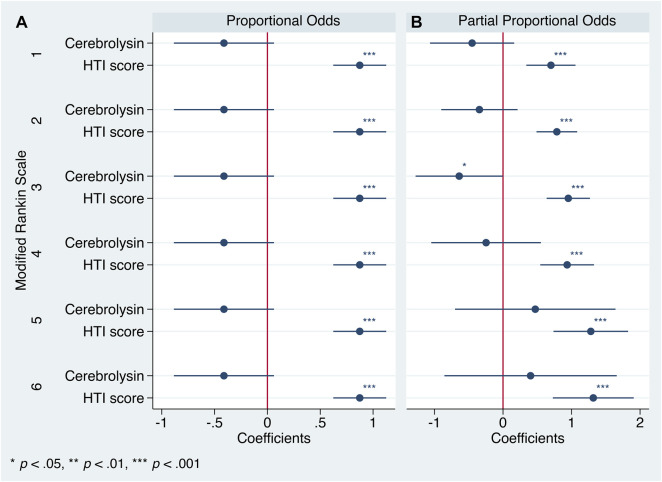
Generalized ordered logistic regression analysis: coefficients with 95% CIs (*n* = 238). mRS = 0 is the base category. **(A)**. Proportional odds assumption. **(B)**. Partial proportional odds assumption.

The Brant test turned out to be insignificant (χ^2^ (10) = 4.31, *p* = 0.932), hence the parallel regression assumption was not violated and generalized ordered logistic regression with proportional as well as partial proportional odds was justified for analysis. Both approaches defined the HTI score as a significant predictor of functional outcome (Figure 5).

**FIGURE 6:**
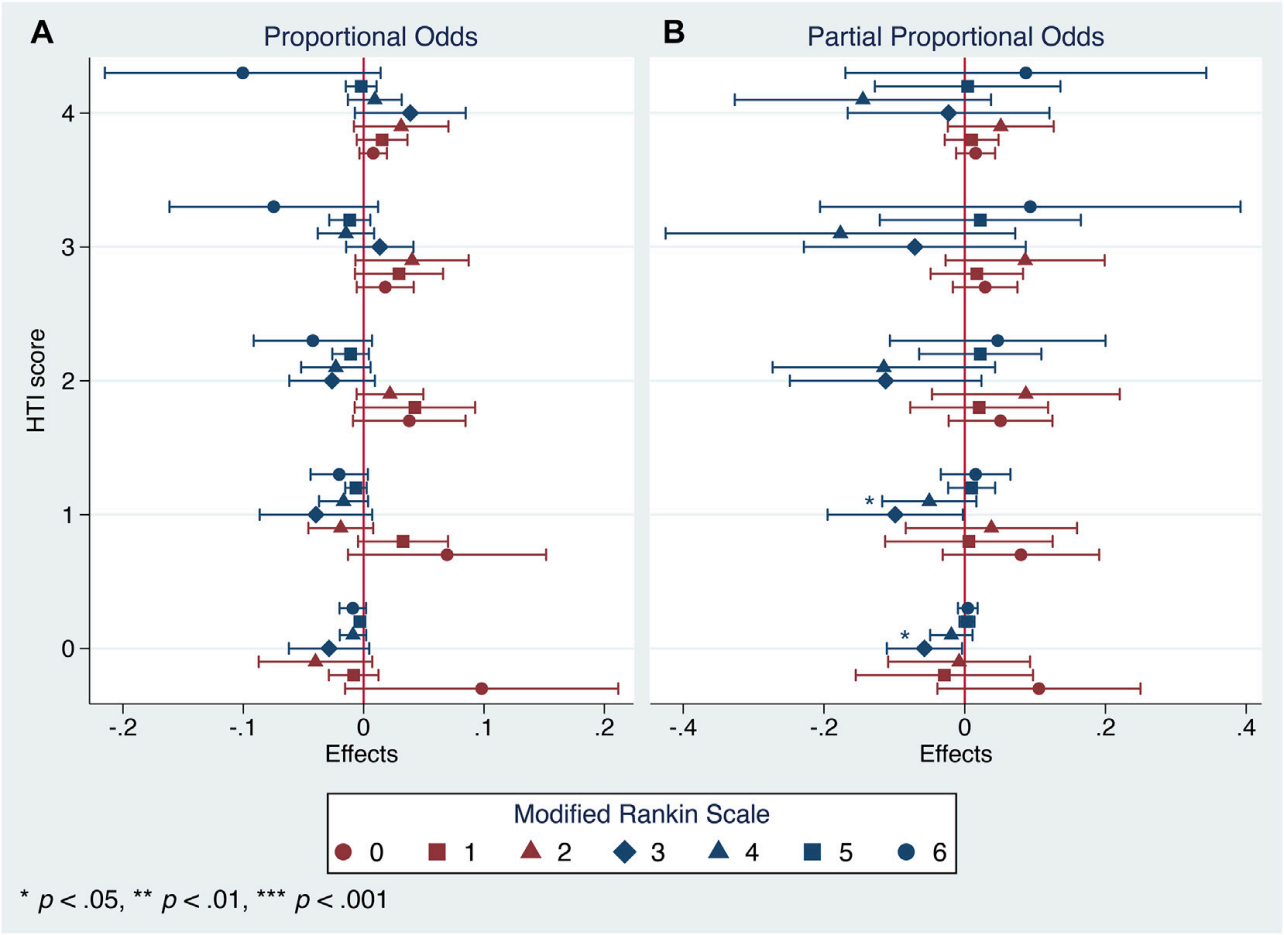
Generalized ordered logistic regression analysis: conditional marginal effects of the Cerebrolysin group on probability of varying mRS scores on day 90 by the HTI score with 95% CIs (*n* = 238). **(A)**. Proportional odds assumption. **(B)**. Partial proportional odds assumption.

After applying the partial proportional odds assumption, the aforementioned first trend did achieve the statistical significance but was concealed by the use of a less flexible technique, the parallel one. However, both procedures failed to provide robust estimates of the second trend (Figures 5, 6).

Although the applied statistical approaches did establish some clinical variability in Cerebrolysin treatment effects among patients with varying HT risk, their heterogeneity was confirmed using methods of meta-analysis. The subgroup of patients with HTI = 4 was omitted during the procedure due to missing values in the Cerebrolysin arm (Table 3).

**FIGURE 7:**
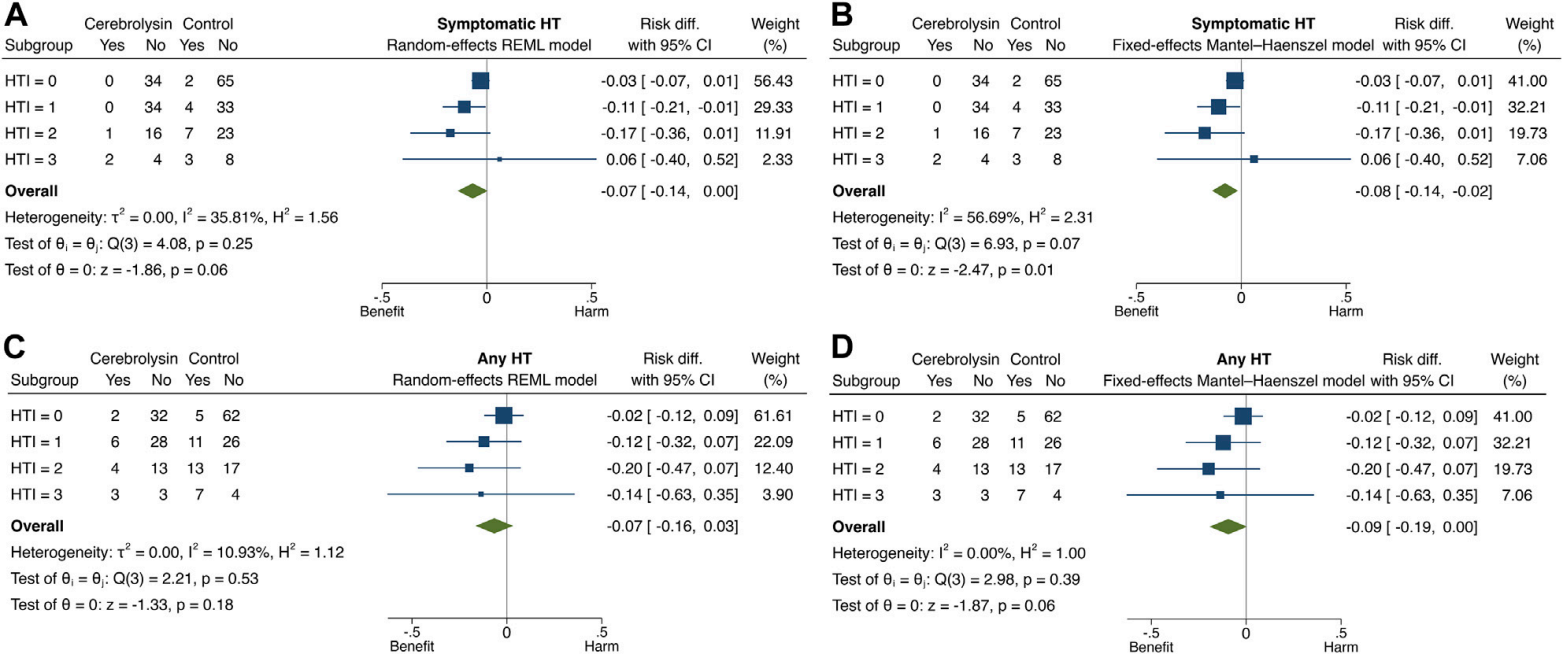
Forest plot and heterogeneity statistics of Cerebrolysin treatment effects on HT (*n* = 236). **(A)**. Symptomatic HT, random-effects restricted maximum likelihood (REML) model. **(B)**. Symptomatic HT, fixed-effects Mantel–Haenszel model. **(C)**. Any HT, random-effects REML model. **(D)**. Any HT, fixed-effects Mantel–Haenszel model.

**FIGURE 8:**
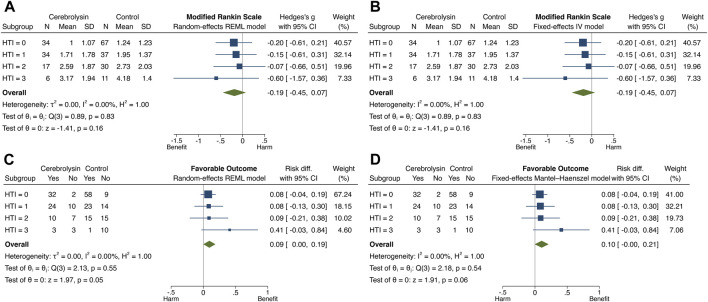
Forest plot and heterogeneity statistics of Cerebrolysin treatment effects on functional outcome (*n* = 236). **(A)**. Modified Rankin Scale (mRS), random-effects restricted maximum likelihood (REML) model. **(B)**. mRS, fixed-effects inverse-variance (IV) model. **(C)**. Favorable functional outcome (FFO), random-effects REML model. **(D)**. FFO, fixed-effects Mantel–Haenszel model.

The overall and subgroup direction and size of Cerebrolysin treatment effects on HT and functional outcome did not differ much between the general population (the random-effects model) and studied cohort (the fixed-effects model) and were coherent with the results of the previous tests. By the way, no small-study effects, the phenomenon that smaller subgroups (“studies”) showed different treatment effects than large ones, were found in any model of interest (Egger test, *p* > 0.05). Heterogeneity of Cerebrolysin treatment effects turned out to be moderate (I^2^, 35.8%–56.7%; H^2^, 1.56–2.31) and mild (I^2^, 10.9%; H^*2*^, 1.12) for symptomatic and any HT, respectively. However, the approach was unrevealing for functional outcome (Figures 7, 8).

**FIGURE 9:**
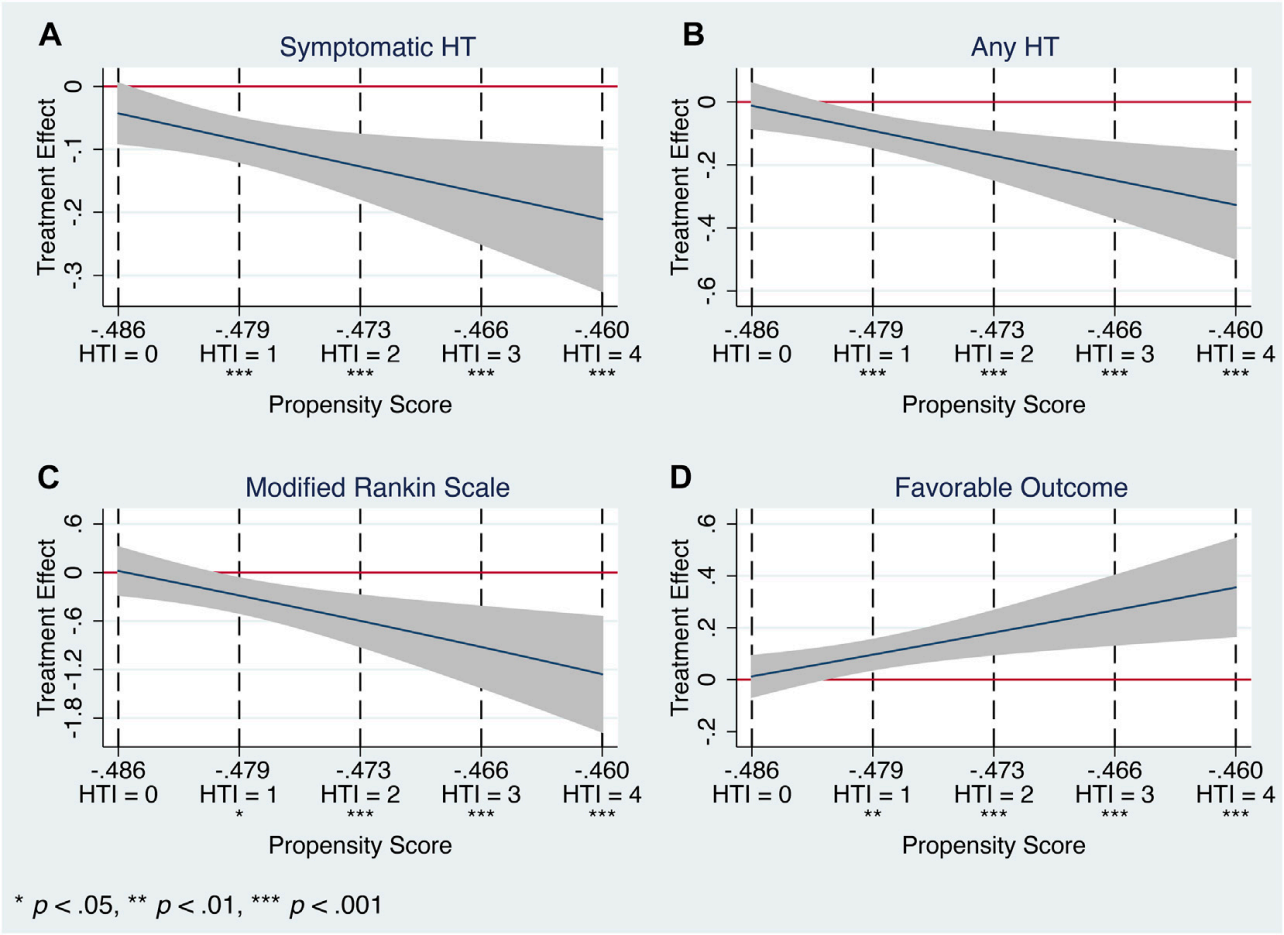
Heterogeneous treatment effects of Cerebrolysin with 95% CIs using the matching-smoothing method (*n* = 238). A local polynomial fit of degree 1 (local-linear smoothing) is used. Each HTI score is matched to the corresponding propensity score with a dashed vertical line. **(A)**. Symptomatic HT. **(B)**. Any HT. **(C)**. Modified Rankin Scale on day 90. **(D)**. Favorable functional outcome.

Nevertheless, HTE analysis by means of the matching-smoothing method demonstrated a significant positive impact of the Cerebrolysin treatment on HT and functional outcome: it was neutral when the risk of HT and poor functional outcome was low (HTI = 0) but gradually became evident in the moderate (HTI = 1) and high (HTI ≥2) HT risk patients. Largely, Cerebrolysin HTEs appeared in a linear-slope manner (Figure 9).

In particular, there was a steady decline in the rate of symptomatic (HTI = 0 vs. HTI = 4: by 4.3%, *p* = 0.077 vs. 21.1%, *p* < 0.001, respectively) and any HT (HTI = 0 vs. HTI = 4: by 1.2%, *p* = 0.737 vs. 32.7%, *p* < 0.001, respectively). Likewise, Cerebrolysin treatment resulted in an overall reduction of the mRS score, which was significantly greater with increasing HTI scores (HTI = 0 vs. HTI = 4: by 1.8%, *p* = 0.903 vs. 126%, *p* < 0.001, respectively). Reciprocally, a growing fraction of patients with FFO (HTI = 0 vs. HTI = 4: by 1.2% *p* = 0.757 vs. 35.5%, *p* < 0.001, respectively) was observed with climbing HTI scores (Table 5; Figure 9).

**TABLE 5:** HTE analysis using the matching-smoothing method (*n* = 238).

HTI score	Propensity score	Symptomatic HT		Any HT		mRS on day 90		Favorable outcome	
		TE (95% CI)	*p*-value	TE (95% CI)	*p*-value	TE (95% CI)	*p*-value	TE (95% CI)	*p*-value
0	−0.486	−0.043 (−0.090, 0.005)	0.077	−0.012 (−0.083, 0.059)	0.737	0.018 (−0.276, 0.313)	0.903	0.012 (−0.066, 0.091)	0.757
1	−0.479	−0.084 (−0.119, −0.050)	<0.001	−0.090 (−0.142, −0.038)	<0.001	−0.279 (−0.493, −0.065)	0.011	0.094 (0.037, 0.152)	0.001
2	−0.473	−0.126 (−0.175, −0.076)	<0.001	−0.174 (−0.251, −0.096)	<0.001	−0.612 (−0.933, −0.290)	<0.001	0.185 (0.099, 0.271)	<0.001
3	−0.466	−0.170 (−0.251, −0.089)	<0.001	−0.251 (−0.371, −0.130)	<0.001	−0.929 (−1.432, −0.426)	<0.001	0.269 (0.135, 0.403)	<0.001
4	−0.460	−0.211 (−0.325, −0.097)	<0.001	−0.327 (−0.496, −0.158)	<0.001	−1.260 (−1.969, −0.551)	<0.001	0.355 (0.168, 0.543)	<0.001

CI, confidence interval; mRS, modified Rankin Scale; TE, treatment effect.

Thus, eligible for IVT patients with estimated on-admission HT risk of the moderate or high grade turned out to be the most responsive to the Cerebrolysin treatment.

## Discussion

Using sophisticated statistical analysis, our *post hoc* study established clinically meaningful heterogeneity of Cerebrolysin treatment effects with respect to HT mitigation and functional outcome improvement among patients with varying HT risk. More importantly, the positive impact of the Cerebrolysin treatment grew steadily along with increasing HT risk. In general, the study results were coherent with the original report (Khasanova and Kalinin, 2023) and discovered a group of patients benefiting most by the proposed treatment.

Treatment effect refers to the causal effect of a treatment or intervention on an outcome of interest based on the counterfactuals (e.g., difference in outcomes with/without using the drug). HTE analysis focuses on assessing varying treatment effects for individuals or subgroups in a population (Gong et al., 2021).

Estimation of HTEs plays an essential role in randomized clinical trials, in particular, to identify patients who benefit most by the intervention for developing personalized treatment (Imai and Ratkovic, 2013; Ling et al., 2023). Meanwhile, traditional statistical approaches like descriptive statistics and regression analysis have a strong tendency to focus on the significance of the estimated overall ATE rather than systematically evaluate the variation in treatment effects across subgroups. As a result, there has been the standard practice to report a number of single estimates representing the treatment efficacy (Imai and Strauss, 2011).

HTEs are usually examined with a predictive model for individual outcomes followed by the exploration of interactions between treatment allocation and important patients’ baseline characteristics. In contrast, our HTE analysis was in favor of the two-stage approach (Watson and Holmes, 2020) since it allowed us to avoid overfitting and transformations of the outcome measurements.

Moreover, assessment of HTEs could especially be useful in settings in which univariate subgroup analyses are unrevealing (Raghavan et al., 2022). For instance, heterogeneity of treatment effects alone may be considered as a sufficient explanation for negative results in studies of some neuroprotective agents because even in the largest trials sample size is inadequate to detect effect size equivalent to those with IVT (Samsa and Matchar, 2001; Muir, 2002). Indeed, various regression approaches failed to provide robust estimates of the functional outcome improvement in our study, whereas HTE analysis did unambiguously confirm those patterns.

However, HTE analysis has not been widely used despite the fact that treatment effects are rarely perfectly homogeneous over the population. Perhaps, part of the reason is the complexity of its methods (Gong et al., 2021). Indeed, only few studies addressing HTEs in stroke patients have been published by now (Kent et al., 2001; Kent et al., 2021; Zhou et al., 2022; Ngufor et al., 2023; Zhu et al., 2023), but none of them related to neuroprotection. Therefore, we have striven to adhere meticulously to the proposed guidelines (Gabler et al., 2009) to report the results of our HTE analysis.

A number of tools have been suggested to predict HT in stroke patients (Kalinin et al., 2017). Although many of them are reliable and composed of the same set of predictors with some variations, only the HTI score takes into account a vascular territory of the infarcted brain lesions. This could be a reason why the HTI score outperformed the DRAGON and SEDAN scores in HT risk stratification in MCA stroke patients.

There are numerous well-established differences between stroke in the posterior and anterior circulation (Libman et al., 2001; Sarikaya et al., 2011; Merwick and Werring, 2014), which could be a source of additional heterogeneity of treatment effects. Moreover, some HT risk assessment tools turned out to be of little value in patients with posterior circulation stroke (Sung et al., 2013). Therefore, only MCA stroke patients were included in the study because we considered them to a certain extent as a homogeneous cohort from the anatomical point of view.

Permeability–surface area product (PS) is a known perfusion imaging marker of BBB permeability and an independent HT predictor. The HTI score is able to predict not only the HT probability but also brain perfusion data, in particular, PS values. The more PS rises in the infarct core following stroke, the higher the HTI score, and hence, the higher the HT risk (Kalinin et al., 2019).

Moreover, rtPA increases the HT rate by degrading the BBB integrity and promoting neuroinflammation and excitotoxicity (Wang et al., 2004; Kelly et al., 2006; Guan et al., 2019). On the other hand, Cerebrolysin ameliorates rtPA adverse effects (Veinbergs et al., 2000; Teng et al., 2021; Guan et al., 2019), and our original report has confirmed neuroprotective and BBB stabilizing features of Cerebrolysin in clinical settings: an improvement in the diffusion-tensor imaging metrics and PS was observed in the infarcted lesions (Khasanova and Kalinin, 2023). Therefore, benefiting most by the Cerebrolysin treatment in HT-risky circumstances could be explained by more efficient salvage of the ischemic brain tissue as well as more prominent attenuation of BBB permeability and mitigation of rtPA adverse effects with the Cerebrolysin use.

Based on high-quality evidence, current clinical guidelines strongly recommend IVT for patients with acute ischemic stroke of <4.5 h duration (Berge et al., 2021). Since the earliest publications of systematic reviews and meta-analyses on IVT-related HT, a paradigm that no reliable method can provide the individualization of treatment according to predicted HT risk has dominated (Whiteley et al., 2012), which practically makes all available prediction tools useless. As a result, prior HT risk assessment is not even required to start IVT in stroke patients. In that respect, our research is one of the first steps to shift that paradigm.

Several clinical studies have investigated the concomitant use of various agents, which reduce the HT rate and exert multimodal effects, alongside IVT. However, phase III clinical trials are required to confirm the observed positive results (Otsu et al., 2020). The ESCAPE-NA1 trial, a large-scale study of the neuroprotective agent nerinetide, failed to demonstrate an improvement in functional outcome due to a possible drug-drug interaction with alteplase (Hill et al., 2020). In that respect, Cerebrolysin could be an alternative to nerinetide and other candidates given its ability to mitigate rtPA-related adverse effects, well-established safety profile and long-lasting use in clinical practice worldwide.

Clinical practitioners frequently encounter so called the risk-treatment paradox, in which patients at the highest risk (and with the greatest potential to gain from the treatment) are treated less often than those at lower risk and with less potential to benefit (Spertus et al., 2015). In that respect, our study is another step towards integrating individualized risk stratification within routine clinical practice to disregard this misconception, to remind clinicians of each patient’s potential benefits from the treatment and to ensure more patient-orientated, evidence-based care with favorable outcomes.

The strength of the study emerged from stratification of stroke patients according to their HT risk followed by a complex statistical approach to analyze heterogeneity of Cerebrolysin treatment effects.

Nevertheless, there are a few limitations in our study. First, an anatomical restriction: only MCA stroke patients were selected for analysis. Heterogeneity of treatment effects in subjects with vertebrobasilar infarction can significantly affect the outcome of interest and requires a separate subanalysis.

Second, an incomplete range of the HTI score: due to study exclusion criteria, the population with an HTI score of ≤4 was only analyzed. Obviously, patients with an HTI score beyond this limit would fall into a category of extremely high risk of HT and would have a large infarct core and severe stroke. In those circumstances, IVT might be considered in some selected cases based on the results of advanced brain imaging (core/perfusion mismatch) and other contraindications (Berge et al., 2021). In that respect, a clinical trial on patients with severe stroke and futile recanalization after IVT has suggested Cerebrolysin as an add-on to IVT is safe and reduces the HT and mortality rate (Poljakovic et al., 2021). Moreover, recent clinical trials on mechanical endovascular thrombectomy (EVT) in patients with a large infarct core (Yoshimura et al., 2022; Huo et al., 2023; Sarraj et al., 2023) have shown a significant improvement in functional outcome compared with the standard medical care alone despite an increased HT rate in the intervention group. Thus, it is fairly reasonable to assume the Cerebrolysin treatment combined with reperfusion therapy could result in a reduction of the HT rate and further enhancement of functional outcome in those patients.

At last, *post hoc* analyses inherently suffer from a well-known statistical problem: the subgroups are formed after the trial is conducted, and such analyses bear a risk of finding statistically significant results when no true relationship exists (Imai and Strauss, 2011). In that respect, there are a number of challenges related to researcher bias in secondary data analysis such as prior knowledge of data, non-hypothesis-driven research, inappropriateness and lack of flexibility in data analysis (Baldwin et al., 2022). Therefore, a variety of approaches were applied to address this issue (Table 6). Yet, further research with prespecified HT risk subgroups is required.

**TABLE 6:** Research bias and approaches to overcome it in *post hoc* data analysis.

Challenge	Potential solution (Baldwin et al., 2022)	Comments
Prior knowledge of data	Declaring prior access to data; conducting multiverse analysis	There was prior access to the dataset. All potential analytic approaches were identified, justifiably implemented to address a given research question, and the results were reported
Non-hypothesis-driven research	Pre-formulating a research question and conditions for interpretation; using a hold-out sample to delineate exploratory and confirmatory research	A specific, concise and testable hypothesis was stated prior to secondary data analysis. The sample size was insufficient for splitting the dataset, and bootstrapping was used to reduce sampling bias
Inappropriateness in data analysis	Trial analysis on a blinded dataset; trial analysis on a dataset without outcome measures; creating a decision tree	The sandbox datasets were not available because of prior knowledge of the data and a relatively small sample size. A number of tests were performed to ensure data suitability for analysis such as validation for missing values, specification errors, nonlinearity, collinearity, goodness of fit, proportional odds assumption, small-study effects, and adjustment for multiple hypothesis testing and multiple comparisons. The study flow-chart was also provided
Lack of flexibility in data analysis	Transparent reporting unplanned analyses; using methods to interpret non-significant results	No methodological deviations from pre-planned data analysis occurred. Statistically significant as well as non-significant findings were reported and discussed. HTE analysis by means of the matching-smoothing method was applied for final interpretation of the results

### Recommendations for clinical practice

**FIGURE 10:**
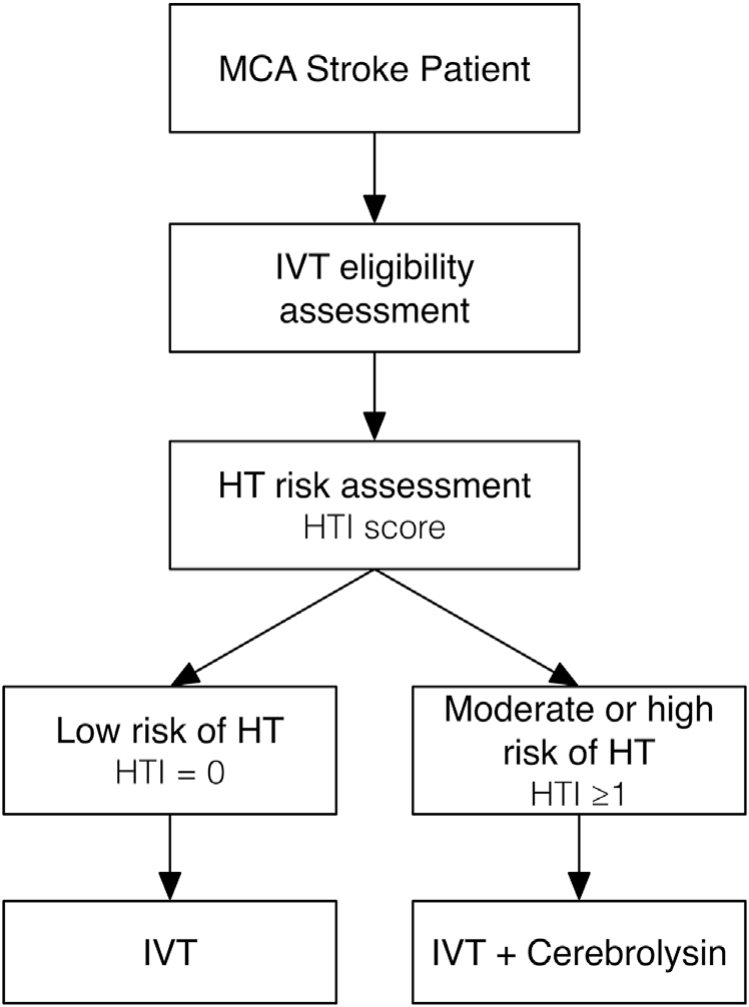
A flow-chart of the proposed recommendations for clinical practice.

Based on the results of our original research and *post hoc* analysis, recommendations for clinical practice can be proposed as follows (Figure 10):

1. All patients with clinical and imaging data of acute MCA ischemic stroke of <4.5 h duration should be checked against eligibility criteria for IVT.
2. The HT risk assessment with prediction tools like the HTI score should be performed before starting IVT.
3. For IVT-eligible patients with the low (HTI = 0) risk of HT, IVT alone may be considered.
4. For IVT-eligible patients with the moderate (HTI = 1) or high (HTI ≥2) risk of HT, IVT with a concurrent add-on of a neuroprotective agent like Cerebrolysin (intravenous infusion of 30 mL in 100 mL of normal saline via separate line over 20 min) is strongly recommended to prevent early HT.
5. Following IVT, treatment with a neuroprotective agent like Cerebrolysin (the same dose once daily) might be continued for as long as 14 consecutive days to prevent delayed HT and to further enhance functional recovery.

### Future research directions

Nowadays mechanical EVT has become the standard reperfusion therapy for acute ischemic stroke due to large vessel occlusion in the anterior circulation (Powers et al., 2019). Besides early-window (<6 h) reperfusion, EVT is being extensively studied in other clinical settings: prior IVT bridging, late-window (6–24 h) and very late-window (>24 h) time frame, a large infarct core, and subsequent futile recanalization (Manning et al., 2018; Lin et al., 2022; Kobeissi et al., 2023; Sattari et al., 2023; Shen et al., 2023). However, reperfusion injury and BBB disruption leading to HT and poor outcome inevitably occur in some patients. Future research on Cerebrolysin along with EVT and prior HT risk assessment is required in those clinical scenarios. As the first step, an ongoing clinical trial, the efficacy of Cerebrolysin treatment as an add-on therapy to mechanical thrombectomy in patients with acute ischemic stroke due to large vessel occlusion, has already set the rate of symptomatic HT as a secondary endpoint (Staszewski et al., 2022).

In posterior circulation stroke, the HT risk after IVT is half that of anterior one, with similar functional outcomes and higher risk of death, acknowledging limitations of the NIHSS for stroke severity or infarct size adjustment (Sarikaya et al., 2011; Keselman et al., 2020). On the other hand, EVT increases the HT rate but significantly decreases the risk of 90-day mortality (Dong et al., 2022; Xu et al., 2023). Therefore, more research is required to assess the HT risk and the use of Cerebrolysin alongside reperfusion therapy in those clinical settings.

Finally, COVID-19 has had an adverse impact on acute ischemic stroke patients who undergo reperfusion therapy, leading to an elevated risk of HT, higher mortality and lower likelihood of FFO (Stuckart et al., 2023). Hence, further studies can highlight an impact of Cerebrolysin combined with reperfusion therapy in COVID-19 patients.

## Conclusion

Clinically meaningful HTEs of Cerebrolysin as an early add-on to reperfusion therapy were established in patients with varying HT risk. In terms of FFO achievement and HT rate reduction after IVT, the Cerebrolysin treatment appeared to be beneficial in those whose estimated on-admission HT risk was either moderate or high. The evidence is sufficient to guide different treatment recommendations in one or more subgroups but warrants future research with prespecified hypotheses.

## Data Availability

The original contributions presented in the study are included in the article/Supplementary Material, further inquiries can be directed to the corresponding author.

## Abbreviations

ASPECTS, Alberta Stroke Program Early Computed Tomography Score; ATC, average treatment effects on the untreated; ATE, average treatment effects; ATT, average treatment effects on the treated; BBB, blood-brain barrier; CI, confidence interval; DRAGON, (hyper)Dense cerebral artery sign/early infarct signs on admission computed tomography scan, prestroke modified RAnkin scale, Glucose level at baseline, Onset-to-treatment time, baseline NIHSS; EVT, endovascular thrombectomy; FFO, favorable functional outcome; HMCA, hyperdense middle cerebral artery; HT, hemorrhagic transformation; HTE, heterogeneous treatment effect; HTI, Hemorrhagic Transformation Index; IQR, interquartile range; IVT, intravenous thrombolysis; M, median; MCA, middle cerebral artery; mRS, modified Rankin Scale; NATE, naive average treatment effects; NIHSS, National Institutes of Health Stroke Scale; OR, odds ratio; PS, permeability–surface area product; rtPA, recombinant tissue plasminogen activator; SEDAN, baseline blood Sugar, Early infarct signs, (hyper)Dense cerebral artery sign, Age, NIHSS on admission.
