# Supplementary material for: Heterogeneous treatment effects of Cerebrolysin as an early add-on to reperfusion therapy: post hoc analysis of the CEREHETIS trial
Source: Front Pharmacol. 2024 Jan 5;14:1288718. doi: 10.3389/fphar.2023.1288718 (PMC10796496; doi:10.3389/fphar.2023.1288718)
Supplement: Supplementary file 1 [file DataSheet3.docx]

***Supplementary Material***

Front. Pharmacol. 14:1288718. doi: 10.3389/fphar.2023.1288718

**Heterogeneous treatment effects of Cerebrolysin as an early add-on to reperfusion therapy: post hoc analysis of the CEREHETIS trial**

Mikhail N. Kalinin^1,2^ and Dina R. Khasanova^1,2^

1. Department of Neurology, Kazan State Medical University, Kazan, Russia

2. Department of Neurology, Interregional Clinical Diagnostic Center, Kazan, Russia

**Stata code for statistical analysis**

**//introduction**

* files **Data Sheet 1.XLSX** and **Date Sheet 2.XLSX** should be imported using StataMP v.17 or above and converted into **.dta**-format

* this code can be copied and pasted in a new Stata **.do**-file for easy execution

**//defining variables from file Data Sheet 2.dta**

* rid – randomization id number

* sht - symptomatic HT (yes=1, no=0)

* anyht - any HT (yes=1, no=0)

* ffo - favorable functional outcome (yes=1, no=0)

* group - allocation group (Cerebrolysin=1, Control=0)

* mrs - mRS score on day 90

* hti - HTI score

* dragon - DRAGON score

* sedan - SEDAN score

**//additional packages to be downloaded**

* use command **findit** to find corresponding package from the repository:

* **coefplot**

* **grc1leg**

* **boxtid**

* **fitstat**

* **roccomp**

* **collin**

* **rwolf2**

* **mplotoffset**

* **gologit2**

* **kmatch**

* **hte**

* **transform_margins**

**net from** http://www.stata.com/users/jpitblado

**net describe** transform_margins

**net install** transform_margins

**help** transform_margins

**//analysis starts**

**use** "Data Sheet 2.dta", clear

**//figue 2, table 2: OR, postestimation tests, model selection:**

*table 2:

**model 1; sympt HT: DRAGON + Cerebrolysin and postestimation tests

**logit** sht group dragon, vce (bootstrap, reps(1000) bca ties nodots seed(2000000)) nolog or

**est store** sht_dra

**predict** p1 if e(sample), p

**estat gof**, group(10)

**fitstat**

**linktest**, nolog

**boxtid** logit sht group dragon, nolog

**model 2; sympt HT: SEDAN + Cerebrolysin and postestimation tests

**logit** sht group sedan, vce (bootstrap, reps(1000) bca ties nodots seed(2000000)) nolog or

**est store** sht_sed

**predict** p2 if e(sample), p

**estat gof**, group(10)

**fitstat**

**linktest**, nolog

**boxtid** logit sht group sedan, nolog

**model 3; sympt HT: HTI + Cerebrolysin and postestimation tests

**logit** sht group hti, vce (bootstrap, reps(1000) bca ties nodots seed(2000000)) nolog or

**est store** sht_hti

**predict** p3 if e(sample), p

**estat gof**, group(10)

**fitstat**

**linktest**, nolog

**boxtid** logit sht group hti, nolog

**model 4; sympt HT: DRAGON + SEDAN + HTI + Cerebrolysin and postestimation tests

**logit** sht group dragon sedan hti, vce (bootstrap, reps(1000) bca ties nodots seed(2000000)) nolog or

**est store** sht_comb

**predict** p4 if e(sample), p

**estat gof**, group(10)

**fitstat**

**linktest**, nolog

**boxtid** logit sht group dragon sedan hti, nolog

**Likelihood ratio test

**lrtest** sht_comb sht_dra

**lrtest** sht_comb sht_sed

**lrtest** sht_comb sht_hti

**AUC CI and AUC comparison

**roccomp** sht p1 p2 p3 p4

**roccomp** sht p1 p4

**roccomp** sht p2 p4

**roccomp** sht p3 p4

**drop** p1 p2 p3 p4

**test for collinearity

**collin** group dragon

**collin** group sedan

**collin** group hti

**collin** group dragon sedan hti

**Romano-Wolf multiple hypothesis testing, p-value

**rwolf2** (logit sht group dragon) (logit sht group sedan) (logit sht group hti) (logit sht group dragon sedan hti), indepvars (group, group, group, group) seed (2000000) usevalid reps (1000) nodots

*generating figure 2:

**computing OR for any HT and FFO for cerebrolysin + dragon, sedan, hti and their combination:

**logit** anyht group dragon, vce (bootstrap, reps(1000) bca ties nodots seed(2000000)) nolog or

**est store** aht_dra

**logit** ffo group dragon, vce (bootstrap, reps(1000) bca ties nodots seed(2000000)) nolog or

**est store** ffo_dra

**logit** anyht group sedan, vce (bootstrap, reps(1000) bca ties nodots seed(2000000)) nolog or

**est store** aht_sed

**logit** ffo group sedan, vce (bootstrap, reps(1000) bca ties nodots seed(2000000)) nolog or

**est store** ffo_sed

**logit** anyht group hti, vce (bootstrap, reps(1000) bca ties nodots seed(2000000)) nolog or

**est store** aht_hti

**logit** ffo group hti, vce (bootstrap, reps(1000) bca ties nodots seed(2000000)) nolog or

**est store** ffo_hti

**logit** anyht group dragon sedan hti, vce (bootstrap, reps(1000) bca ties nodots seed(2000000)) nolog or

**est store** aht_comb

**logit** ffo group dragon sedan hti, vce (bootstrap, reps(1000) bca ties nodots seed(2000000)) nolog or

**est store** ffo_comb

**adding arrows to figure 2:

**gen** start_2 = .8526905

**gen** start_3 = .714449

**gen** end = 3

**gen** which_3 = 1.25

**gen** start_4 = .6078821

**gen** which_4 = 1.75

**gen** start_5 = 1.0944

**gen** which_5 = 2

**gen** start_10 = 1.15032

**gen** which_10 = 3.75

**gen** start_11 = 1.188142

**gen** which_11 = 4

**gen** start_30 = .8503465

**gen** start_40 = 1.645208

**gen** start_50 = 1.716096

**generating figure 2A:

**coefplot** sht_dra aht_dra ffo_dra, drop (_cons) xline(1) eform xtitle(Odds ratio) mlabposition(1) mlabel(cond(@pval<.001, "***", cond(@pval<.01, "**", cond(@pval<.05, "*", "")))) note("* {it:p} < .05, ** {it:p} < .01, *** {it:p} < .001") transform(* = min(max(@,0),3)) name(g7)

**generating figure 2B:

**coefplot** sht_sed aht_sed ffo_sed, drop (_cons) xline(1) eform xtitle(Odds ratio) mlabposition(1) mlabel(cond(@pval<.001, "***", cond(@pval<.01, "**", cond(@pval<.05, "*", "")))) note("* {it:p} < .05, ** {it:p} < .01, *** {it:p} < .001") transform(* = min(max(@,0),3)) addplot((pcarrow which_3 start_2 which_3 end)) name(g8)

**generating figure 2C:

**coefplot** sht_hti aht_hti ffo_hti, drop (_cons) xline(1) eform xtitle(Odds ratio) mlabposition(1) mlabel(cond(@pval<.001, "***", cond(@pval<.01, "**", cond(@pval<.05, "*", "")))) note("* {it:p} < .05, ** {it:p} < .01, *** {it:p} < .001") transform(* = min(max(@,0),3)) addplot((pcarrow which_3 start_30 which_3 end)(pcarrow which_4 start_40 which_4 end)(pcarrow which_5 start_50 which_5 end)) name(g9)

**generating figure 2D:

**coefplot** sht_comb aht_comb ffo_comb, drop (_cons) xline(1) eform xtitle(Odds Ratio) transform(* = min(max(@,0),3)) addplot((pcarrow which_3 start_3 which_3 end) (pcarrow which_4 start_4 which_4 end) (pcarrow which_5 start_5 which_5 end) (pcarrow which_10 start_10 which_10 end) (pcarrow which_11 start_11 which_11 end)) mlabposition(1) mlabel(cond(@pval<.001, "***", cond(@pval<.01, "**", cond(@pval<.05, "*", "")))) note("* {it:p} < .05, ** {it:p} < .01, *** {it:p} < .001") name(g10)

**combine figure 2A-2D:

**grc1leg** g7 g8 g9 g10, legendfrom(g7)

**dropping saved graphs from memory:

**graph drop** g7 g8 g9 g10

**//Table 4. Predicted probability of symptmatic HT, any HT, and FFO with Sidak corrected 95% CIs**

**logit** sht i.group hti, vce (bootstrap, reps(1000) bca ties nodots seed(2000000)) nolog

**margins** group, at(hti=(0(1)4)) vsquish mcompare(sid) predict(xb)

**transform_margins** invlogit(@)

**logit** anyht i.group hti, vce (bootstrap, reps(1000) bca ties nodots seed(2000000)) nolog

**margins** group, at(hti=(0(1)4)) vsquish mcompare(sid) predict(xb)

**transform_margins** invlogit(@)

**logit** ffo i.group hti, vce (bootstrap, reps(1000) bca ties nodots seed(2000000)) nolog

**margins** group, at(hti=(0(1)4)) vsquish mcompare(sid) predict(xb)

**transform_margins** invlogit(@)

**//figure 3: combine graph - ate att atc nate cme logistic n=238 hti 0-4**

**logit** sht i.group hti, vce (bootstrap, reps(1000) bca ties nodots seed(2000000)) nolog

**est store** sht0

**margins**, dydx(group) at(hti=(0(1)4)) vsquish post

**est store** sht1

**logit** anyht i.group hti, vce (bootstrap, reps(1000) bca ties nodots seed(2000000)) nolog

**est store** aht

**margins**, dydx(group) at(hti=(0(1)4)) vsquish post

**est store** aht1

**logit** ffo i.group hti, vce (bootstrap, reps(1000) bca ties nodots seed(2000000)) nolog

**est store** fout

**margins**, dydx(group) at(hti=(0(1)4)) vsquish post

**est store** fout1

**bootstrap**, reps(1000) bca ties seed(2000000) reject(e(k_omit)) nodots: kmatch ps group hti (sht), ate nate po att atc

**est store** sht3

**bootstrap**, reps(1000) bca ties seed(2000000) reject(e(k_omit)) nodots: kmatch ps group hti (anyht), ate nate po att atc

**est store** aht3

**bootstrap**, reps(1000) bca ties seed(2000000) reject(e(k_omit)) nodots: kmatch ps group hti (ffo), ate nate po att atc

**est store** fout3

**coefplot** sht0 aht fout, xline(0) mlabposition(1) mlabgap(*2) drop(_cons) mlabel(cond(@pval<.001, "***", cond(@pval<.01, "**", cond(@pval<.05, "*", "")))) note("* {it:p} < .05, ** {it:p} < .01, *** {it:p} < .001") name(g1)

**coefplot** sht1 aht1 fout1, ciopts(recast(rcap)) xline(0) mlabposition(1) mlabel(cond(@pval<.001, "***", cond(@pval<.01, "**", cond(@pval<.05, "*", "")))) note("* {it:p} < .05, ** {it:p} < .01, *** {it:p} < .001") name(g2)

**coefplot** sht3 aht3 fout3, ciopts(recast(rcap)) xline(0) mlabposition(1) mlabel(cond(@pval<.001, "***", cond(@pval<.01, "**", cond(@pval<.05, "*", "")))) note("* {it:p} < .05, ** {it:p} < .01, *** {it:p} < .001") name(g3)

**grc1leg** g1 g2 g3, cols(3) legendfrom(g1)

*dropping saved graphs from memory:

**graph drop** g1 g2 g3

**//figure 4: mRS by HTI score**

*generating group variables - number of pts per each HTI category over group

**gen** g1 = 1 if group==1

**replace** g1 = 0 if group==0

**gen** g2 = 1 if group==1 & hti==0

**replace** g2 = 0 if group==0 & hti==0

**gen** g3 = 1 if group==1 & hti==1

**replace** g3 = 0 if group==0 & hti==1

**gen** g4 = 1 if group==1 & hti==2

**replace** g4 = 0 if group==0 & hti==2

**gen** g5 = 1 if group==1 & hti==3

**replace** g5 = 0 if group==0 & hti==3

**gen** g6 = 1 if group==1 & hti==4

**replace** g6 = 0 if group==0 & hti==4

**gen** g7 = 1 if group==1

**replace** g7 = 0 if group==0

*generating the graph

**loc** i = 1

**foreach** j in g1 g2 g3 g4 g5 g6 g7 {

**options for graph

**if** `i' == 7 loc legg `"legend(on pos(7) row(1) region(lcolor(none)))"'

**if** `i' !=7 loc axis `"ylabel(none, nolabels nogrid) "'

**if** `i' == 7 loc axis `"yla(0(25)100, nogrid) "'

**lab var** mrs `"`=upper("`j'")'"'

**graph

**catplot** mrs, over(`j') stack percent(`j') asyvars legend(off) `legg' blabel(bar, format(%3.0f) size(small) pos(center) color(gs12)) name(g`i', replace) `axis' plotregion(fcolor(none) lcolor(none)) l1title("`=upper("`j'")'", margin(0 `diff' 0 0 )) ytitle("") yscale(noline) yline(25 50 75 100, extend)

**loc plots** `"`plots' g`i' "'

**loc** `++i'

**}**

**gr combine** `plots', colfirst ycommon cols(1) imargin(zero) graphregion(margin(large))

**drop** g1 g2 g3 g4 g5 g6 g7

**//ordered logistic regression - testing for parallel assumption - mRS by HTI**

**ologit** mrs i.group hti, or nolog

* testing for paralell assumption

**oparallel**

**brant**

**//figure 5: GOL - coeff plot gologit2 n = 238, HTI 0-4:**

**gologit2** mrs i.group hti, pl

**est store** mrs1

**gologit2** mrs i.group hti, npl

**est store** mrs2

**coefplot** mrs1 || mrs2, keep(*:) drop(_cons) xline(0) mlabposition(1) mlabel(cond(@pval<.001, "***", cond(@pval<.01, "**", cond(@pval<.05, "*", "")))) note("* {it:p} < .05, ** {it:p} < .01, *** {it:p} < .001") byopts(xrescale)

**//figure 6: GOL - CME plot gologit2 n =238 HTI =0-4**

**gologit2** mrs i.group hti, pl

**margins**, dydx(group) at(hti=(0(1)4)) vsquish post

**mplotoffset**, offset(0.1) recast(scatter) horiz xline(0) legend(row(1)) name(g1)

**gologit2** mrs i.group hti, npl

**margins**, dydx(group) at(hti=(0(1)4)) vsquish post

**mplotoffset**, offset(0.1) recast(scatter) horiz xline(0) legend(row(1)) name(g2)

**grc1leg** g1 g2, cols(3) legendfrom(g1)

*dropping saved graphs from memory:

**graph drop** g1 g2

**//figure 9: Combine graph HTE HTI:**

**hte ms** sht group hti, logit kernel odds lpolyci(degree(1)) yline(0) xline( -.4856067) xline( -.479201 ) xline (-.4727953) xline( -.4663896) xline( -.4599838) xlabel(-.4856067 `" "-.486" "HTI = 0" "' -.479201 `" " -.479" "HTI = 1" "' -.4727953 `" " -.473" "HTI = 2" "' -.4663896 `" "-.466" "HTI = 3" "' -.4599838 `" "-.460" "HTI = 4" "' ) noscatter legend(off) name(g1)

**hte ms** anyht group hti, logit kernel odds lpolyci(degree(1)) yline(0) xline( -.4856067) xline( -.479201 ) xline (-.4727953) xline( -.4663896) xline( -.4599838) xlabel(-.4856067 `" "-.486" "HTI = 0" "' -.479201 `" " -.479" "HTI = 1" "' -.4727953 `" " -.473" "HTI = 2" "' -.4663896 `" "-.466" "HTI = 3" "' -.4599838 `" "-.460" "HTI = 4" "' ) noscatter legend(off) name(g2)

**hte ms** mrs group hti, logit kernel odds lpolyci(degree(1)) yline(0) xline( -.4856067) xline( -.479201 ) xline (-.4727953) xline( -.4663896) xline( -.4599838) xlabel(-.4856067 `" "-.486" "HTI = 0" "' -.479201 `" " -.479" "HTI = 1" "' -.4727953 `" " -.473" "HTI = 2" "' -.4663896 `" "-.466" "HTI = 3" "' -.4599838 `" "-.460" "HTI = 4" "' ) noscatter legend(off) name(g3)

**hte ms** ffo group hti, logit kernel odds lpolyci(degree(1)) yline(0) xline( -.4856067) xline( -.479201 ) xline (-.4727953) xline( -.4663896) xline( -.4599838) xlabel(-.4856067 `" "-.486" "HTI = 0" "' -.479201 `" " -.479" "HTI = 1" "' -.4727953 `" " -.473" "HTI = 2" "' -.4663896 `" "-.466" "HTI = 3" "' -.4599838 `" "-.460" "HTI = 4" "' ) noscatter legend(off) name(g4)

**grc1leg** g1 g2 g3 g4, rows(2) legendfrom(g1)

**graph drop** g1 g2 g3 g4

*matching ps score with HTI score

**tab** _pscore hti

* from the tabulation table:

* hti = 0, ps score = -.4856067

* hti = 1, ps score = -.479201

* hti = 2, ps score = -.4727953

* hti = 3, ps score = -.4663896

* hti = 4, ps score = -.4599838

**//Table 5: extracting data from the hte graph**

**hte ms** sht group hti, logit kernel odds lpolyci(degree(1)) noscatter legend(off)

**graph save** Graph "Graph.gph", replace

**clear**

**graph use** Graph

**serset dir**

**serset use**

**sum**

*you'll see list of 4 variables:

*use stata data editor to browser the data

*1st variable looks like "__000001"; this is ps score

*2nd variable looks like "__000000"; this is treatment effect, mean value

*3rd variable looks like "__000002"; this is lower bound of 95% confidence interval

*4th variable looks like "__000003"; this is upper bound of 95% confidence interval

*find ps score (see above) matched with HTI score, and copy values of corresponding treatment effect and its CI bounds

**clear**

**use** "Data Sheet 2.dta"

**hte ms** anyht group hti, logit kernel odds lpolyci(degree(1)) noscatter legend(off)

**graph save** Graph "Graph.gph", replace

**clear**

**graph use** Graph

**serset dir**

**serset use**

**sum**

*you'll see list of 4 variables:

*use stata data editor to browser the data

*1st variable looks like "__000001"; this is ps score

*2nd variable looks like "__000000"; this is treatment effect, mean value

*3rd variable looks like "__000002"; this is lower bound of 95% confidence interval

*4th variable looks like "__000003"; this is upper bound of 95% confidence interval

*find ps score (see above) matched with HTI score, and copy values of corresponding treatment effect and its CI bounds

**clear**

**use** "Data Sheet 2.dta"

**hte ms** mrs group hti, logit kernel odds lpolyci(degree(1)) noscatter legend(off)

**graph save** Graph "Graph.gph", replace

**clear**

**graph use** Graph

**serset dir**

**serset use**

**sum**

*you'll see list of 4 variables:

*use stata data editor to browser the data

*1st variable looks like "__000001"; this is ps score

*2nd variable looks like "__000000"; this is treatment effect, mean value

*3rd variable looks like "__000002"; this is lower bound of 95% confidence interval

*4th variable looks like "__000003"; this is upper bound of 95% confidence interval

*find ps score (see above) matched with HTI score, and copy values of corresponding treatment effect and its CI bounds

**clear**

**use** "Data Sheet 2.dta"

**hte ms** ffo group hti, logit kernel odds lpolyci(degree(1)) noscatter legend(off)

**graph save** Graph "Graph.gph", replace

**clear**

**graph use** Graph

**serset dir**

**serset use**

**sum**

*you'll see list of 4 variables:

*use stata data editor to browser the data

*1st variable looks like "__000001"; this is ps score

*2nd variable looks like "__000000"; this is treatment effect, mean value

*3rd variable looks like "__000002"; this is lower bound of 95% confidence interval

*4th variable looks like "__000003"; this is upper bound of 95% confidence interval

*find ps score (see above) matched with HTI score, and copy values of corresponding treatment effect and its CI bounds

**clear**

**use** "Data Sheet 2.dta"

**//Table 5: calculating P-value from confidence intervals:**

**di** "se = " ([ul] - [ll])/(2*1.96)

**di** "z = " [est] / [se]

* go to online p-value calculator at: https://www.graphpad.com/quickcalcs/pValue1/

* use option "p from z-score" to calculate p-value

* use computed z-score to obtain p-value

**clear**

**//Figure 7 and 8: forest plot, heterogeneity of treatment effects**

**defining variables from file Data Sheet 1.dta:

* cat = hti category (like HTI=0,1,2,3)

* tnum_cer = total number of pts, cerebrolysin

* tnum_cont = total number of pts, control

* sht_cer = sympt ht, cerebrolysin

* sht_cont = sympt ht, control

* nosht_cer = no sympt ht, cerebrolysin

* nosht_cont = no sympt ht, control

* anyht_cer = any ht, cerebrolysin

* anyht_cont = any ht, control

* noanyht_cer = no any ht, cerebrolysin

* noanyht_cont = no any ht, control

* ffo_cer = ffo, cerebrolysin

* ffo_cont = ffo, control

* noffo_cer = no ffo, cerebrolysin

* noffo_cont = no ffo, control

* mean_cer = mean, cerebrolysin

* sd_cer = sd, cerebrolysin

* mean_cont = mean, control

* sd_cont = sd, control

**use** "Data Sheet 1.dta"

**meta esize** sht_cer nosht_cer sht_cont nosht_cont, esize(rdiff) studylabel(cat)

**meta forestplot**, random(reml) columnopts(_id, title(Subgroup)) columnopts(_data1, supertitle(Cerebrolysin)) nullrefline

**meta forestplot**, fixed(mhaenszel) columnopts(_id, title(Subgroup)) columnopts(_data1, supertitle(Cerebrolysin)) nullrefline

**meta bias**, egger random(reml)

**meta bias**, egger fixed

**meta esize** anyht_cer noanyht_cer anyht_cont noanyht_cont, esize(rdiff) studylabel(cat)

**meta forestplot**, random(reml) columnopts(_id, title(Subgroup)) columnopts(_data1, supertitle(Cerebrolysin)) nullrefline

**meta forestplot**, fixed(mhaenszel) columnopts(_id, title(Subgroup)) columnopts(_data1, supertitle(Cerebrolysin)) nullrefline

**meta bias**, egger random(reml)

**meta bias**, egger fixed

**meta esize** ffo_cer noffo_cer ffo_cont noffo_cont, esize(rdiff) studylabel(cat)

**meta forestplot**, random(reml) columnopts(_id, title(Subgroup)) columnopts(_data1, supertitle(Cerebrolysin)) nullrefline

**meta forestplot**, fixed(mhaenszel) columnopts(_id, title(Subgroup)) columnopts(_data1, supertitle(Cerebrolysin)) nullrefline

**meta bias**, egger random(reml)

**meta bias**, egger fixed

**meta esize** tnum_cer mean_cer sd_cer tnum_cont mean_cont sd_cont, studylabel(cat)

**meta forestplot**, random(reml) columnopts(_id, title(Subgroup)) columnopts(_data, format(%5.0g)) columnopts(_data1, supertitle(Cerebrolysin)) nullrefline

**meta forestplot**, fixed(invvariance) columnopts(_id, title(Subgroup)) columnopts(_data, format(%5.0g)) columnopts(_data1, supertitle(Cerebrolysin)) nullrefline

**meta bias**, egger random(reml)

**meta bias**, egger fixed
